# Catestatin as a New Prognostic Marker in Stable Patients with Heart Failure with Reduced Ejection Fraction in Two-Year Follow-Up

**DOI:** 10.1155/2020/8847211

**Published:** 2020-10-01

**Authors:** Łukasz Wołowiec, Daniel Rogowicz, Joanna Banach, Wojciech Gilewski, Władysław Sinkiewicz, Grzegorz Grześk

**Affiliations:** Department of Cardiology and Clinical Pharmacology, Faculty of Health Sciences, Ludwik Rydygier Collegium Medicum in Bydgoszcz, Nicolaus Copernicus University in Toruń, Poland

## Abstract

*Background and Purpose*. The main goal of the study was to assess the usefulness of plasma concentrations of catestatin as a predictor of a composite endpoint (CE): unplanned hospitalization and death for all causes in patients with HFrEF in the midterm follow-up. *Experimental Approach*. The study group consisted of 52 Caucasian patients in NYHA classes II and III. The control group consisted of 24 healthy volunteers. The biomarkers, whose concentration was assessed before and after physical exertion as well as the variability of their concentration under the influence of the physical exertion, were NT-proBNP, troponin T, and catestatin. *Key Results*. During the 24-month follow-up period, 11 endpoints were recorded. The univariate analysis of the Cox proportional hazard model showed a statistically significant effect of all assessed CST concentrations on the occurrence of CE. In the 24-month follow-up, where the starting concentration of catestatin was compared with other recognized prognostic factors in HF, the initial concentration of catestatin showed statistical significance in CE prognosis as the only parameter tested. *Conclusions*. Plasma concentration of catestatin before and after physical exertion is a valuable prognostic parameter in predicting death from all causes and unplanned hospitalization in the group of patients with HFrEF in the 2-year follow-up.

## 1. Introduction

The incidence of heart failure (HF) is constantly increasing, due to the aging of the population and as a result of improved survival after acute myocardial infarction (AMI). The prevalence of HF in developed countries is 1-2%, and it is the most common cause of hospitalization among patients >65 years of age [1]. Along with the development of new HF diagnostic and therapeutic methods, it has become important to define and adapt them to a specific group of patients who will benefit most from innovative methods. It results in the growing role of biomarkers in the risk stratification process within the group of HF patients.

Numerous laboratory findings were found to play an important role in defining the group of HF patients with the most serious prognosis. Natriuretic peptides, hyponatremia, C-reactive protein, melanoma cell adhesion molecule (MCAM), procalcitonin, haemoglobin level, or red blood cell distribution width (RDW) are among the most available ones [2–4]. Nonetheless, the search for a new sensitive and specific risk biomarker is still ongoing.

Catestatin (CST) was discovered in 1997, and recent reports on the importance of CST in cardiovascular diseases suggest a cardioprotective role of this peptide. The precursor to CST is chromogranin A (CgA), which is an acidic hydrophilic protein found mainly in the granules of secretory neuroendocrine cells. CST, apart from chromaffin and noradrenergic cells, is also found in myocardial tissues, where a decrease in CST concentration is found with aging [5].

CST is a 21-amino acid peptide whose inhibitory effect on catecholamine secretion occurs through direct interaction with a nicotinic receptor, reducing the influx of extracellular Na+ ions into pheochromocytes [6]. CST is secreted together with catecholamines and by autocrine action; it inhibits further catecholamine secretion by negative feedback [7–9]. A direct antagonistic effect on beta-adrenergic receptors leading to the diminished hypertensive reaction was also described [10]. Another mechanism of CST cardioprotective properties may be derived from the observed *in vivo* vasodilatation. Beneficial afterload reduction resulting from vasodilatation seems to be multifactorial—histamine-mediated mechanism [11] together with the reduction of reactive oxygen species availability and stimulation of nitric oxide production were described [12].

CST is also known to modify the myocardial response in the Frank-Starling mechanism, which proves that it modulates myocardial function under both basal conditions and increased preload [13]. CST activates the *β*2-ARs-PI3K-eNOs-NO signaling pathway in endocardial endothelial cells which play a role in myocardial remodeling. In view of the above, it can be assumed that the inhibition of the fibrotic process by CST occurs through increased production of NO [14]. Another antihypertrophic mechanism may be the phenomenon of CST blocking the receptor for endothelin 1 [15].

## 2. Aim of the Study

The main aim of our study was to assess the usefulness of determining the plasma catestatin concentration (assessed before and after physical exertion) as a predictor of the complex endpoint: unplanned hospitalization and all-cause mortality in the group of patients with HFrEF in a two-year follow-up.

## 3. Methods

### 3.1. Study Population

The study was conducted in 2016-2019. The study group consisted of 52 Caucasian patients with HFrEF in NYHA class II-III, who were either treated on an outpatient basis or hospitalized in the Cardiology Department at the Nicolaus Copernicus University Collegium Medicum University Hospital No. 2 in Bydgoszcz for planned medical procedures, some of which were on the elective list of patients waiting for heart transplantation. Outpatient patients were under the care of the Cardiology Clinic and Heart Failure Clinic operating at the department. The diagnosis of HF was based on the criteria of the European Society of Cardiology from 2016. All patients enrolled in the study were hemodynamically stable for at least 3 months, without the need for an intravenous infusion of positive inotropic drugs or intravenous diuretic therapy, and received optimal pharmacological treatment for each patient in accordance with the ESC guidelines. The control group consisted of 24 healthy volunteers.

The study protocol was approved by the Bioethical Committee of the Nicolaus Copernicus University in Toruń at the Collegium Medicum in Bydgoszcz (KB 591/2016). Each patient signed an informed consent form after obtaining detailed information about the purpose and scope of the study.

The study inclusion criteria included age over 18 years and LVEF < 40% assessed during the current hospitalization or up to 6 months earlier. The exclusion criteria were acute coronary syndrome, active neoplastic disease, active infection, fever of unknown etiology, autoimmune diseases, corticosteroid therapy, uncompensated endocrine disorders, chronic obstructive pulmonary disease, severe impairment of renal function (eGFR < 30 ml/kg/min), impairment of liver function (INR without oral anticoagulation > 1.5 or total bilirubin > 1.5 mg% or the upper limit of the norm for ALT exceeded 3 times), chronic inflammatory bowel disease, and recent surgery (<3 months).

### 3.2. Organization and Course of the Study

All study participants underwent CPET at the time of inclusion in the study. Before and immediately after the end of the study, approximately 10 ml of peripheral blood was collected from participants through venipuncture in the antecubital fossa. Blood was drawn into the vacutainer system tubes containing ethylenediaminetetraacetic acid (EDTA) and tubes without an anticoagulant. Blood samples were centrifuged at 4°C at 3000 rpm for 20 minutes. The resulting plasma samples were placed in Eppendorf tubes and then frozen at -80°C until the CST determination. The remaining samples from the first collection were used to determine other laboratory parameters necessary for proper qualification for the study and necessary in the routine care and treatment of patients with HFrEF. Blood samples in the control group were obtained identically to the study group. The following tests were performed in the local hospital laboratory in all the participants: complete blood count with a leukocyte interest pattern, plasma NT-proBNP concentration with the “ECLIA” electrochemiluminescence method for Elecsys and cobas e analyzers, plasma C-reactive protein (CRP) concentration with the high sensitivity immunoturbidimetric assay for quantitative in vitro determination of CRP in human serum and plasma in Roche/Hitachi cobas c systems, plasma troponin T (TnT, cardiac troponin T) concentration with the electrochemiluminescence method “ECLIA” for Elecsys and cobas e analyzers, plasma creatinine concentration with a calculation of glomerular filtration rate (eGFR calculated according to the sMDRD formula), fasting glucose, and electrolytes.

CEPT was performed in all study participants according to a protocol selected after a preliminary assessment of exertion tolerance by a 6-minute walk test. All major CPET parameters were assessed during the study: VO2peak (max), VE, VE/VO2, VE/VCO2, VO2AT, and OUES.

Due to the fact that CST is a relatively rarely described marker in clinical trials, and there is no range of concentration standards for the general population, the obtained results of the study group were compared with the control group, which included healthy volunteers. Biomarkers whose concentration was assessed before and after exertion, as well as their concentration variability under the influence of physical exertion, were NT-proBNP, TnT, and CST. The patient observation was carried out by phone every 3 months from the patient's enrollment and covered 24 months.

### 3.3. CST Determination

Plasma CST concentration was determined by an enzyme immunoassay (ELISA) kit from RayBiotech® (Norcross, GA, USA), catalog number P10645, dilution factor 12x, reproducibility intra-assay: CV < 10%, and inter-assay: CV < 15%. According to the manufacturer, the reactivity with human CST is 100%. The analytical sensitivity of the method (lower detection limit for the test) is 0.5 ng/ml. The results were read with a LABSYSTEMS iEMS READER MF spectrophotometric reader using the Ascent software version 2.6; the marker was evaluated at 450 nm wavelength. The results were read from the calibration curve prepared for the analyzer used in the study [16].

### 3.4. Statistical Analysis

The results were analyzed using the PQStat software version 1.6.6.202. Analyses were conducted at 0.05 level of significance. Normality was assessed with the Shapiro-Wilk test. In the absence of normal distributions, nonparametric analyses were carried out. Comparisons of quantitative variables in the two groups were conducted with the Student *t*-test (in case of normal distribution in both groups) or with the Mann-Whitney test (otherwise). Sex, HF etiology, NYHA class, eGFR, taking medicines, the presence of arterial hypertension (AHT), DM, atrial fibrillation (AF), or the presence of an implantable cardioverter-defibrillator (ICD) were compared depending on the occurrence of CE during 12 and 24 months of follow-up using Fisher's test. The analysis of two repeated measures was conducted with paired *t*-test (in case of normality of differences) or paired Wilcoxon test (otherwise). A multivariate analysis of the simultaneous impact of many types of drugs on quantitative dependent variables was made by the means of linear regression. 95% confidence intervals were reported along with regression parameters.

The relationship between selected parameters was analyzed by estimating Spearman's rank correlation coefficients. The cutoff points for parameters were established based on the ROC curve. The point on the curve lying closest to the top-left corner of the plot was chosen as a cutoff point. The usefulness of the given parameter as a predictor of the endpoint occurrence was assessed with the area under the ROC curve (AUC). The Kaplan-Meier curves were compared with the log-rank (LR) test. The AUC curves were compared with the DeLong test. The usefulness of combinations of cutoff points for two parameters as predictors of the endpoint occurrence was assessed with sensitivity and specificity. The effect of parameters on the endpoint occurrence was verified using the logistic regression and Cox regression models.

CST *δ*% (variability of CST concentration under the influence of physical effort) was calculated according to the following formula: (CST post (concentration assessed immediately after physical exertion) − CST pre (concentration assessed immediately before physical exertion)) / CST pre × 100.

TnT *δ*% (variability of TnT concentration under the influence of physical effort) was calculated according to the following formula: (TnT post (concentration assessed immediately after physical exertion) − TnT pre (concentration assessed immediately before physical exertion)) / TnT pre × 100.

NT − proBNP *δ*% (variability of NT − proBNP concentration under the influence of physical effort) was calculated according to the following formula: (NT − proBNP − post concentration assessed immediately after physical exertion) −NT − proBNP pre (concentration assessed immediately before physical exertion)) / /NT proBNP pre × 100.

## 4. Results

The study group consisted of 52 Caucasian patients with HFrEF in NYHA class II or III, whose average age was 51.6 ± 9.2 years, and men constituted 90% of the group. Ischemic etiology of heart failure was evident in 30.8% of patients. All patients enrolled in the study were hemodynamically stable without the need for intravenous infusion of positive inotropic drugs and received the optimal pharmacological treatment in accordance with the ESC recommendations. The composite endpoint of the study was unplanned hospitalization and all-cause mortality. During the 12-month follow-up, 6 endpoints were recorded (2 all-cause deaths and 4 unplanned hospitalizations), while in the 24-month period, there were 11 endpoints (2 all-cause deaths and 9 unplanned hospitalizations). During the 12-month follow-up, a statistically significant difference was found in the CST post and CST *δ*% between patients who reached and did not reach CE. Patients who had CE during 24-month follow-up differed statistically significantly from the other patients in respect of all CST levels evaluated (CST pre, CST post, and CST *δ*%), more frequent intake of vitamin K antagonists (VKA), and higher creatinine concentrations. The comparison of the study and the control groups is presented in Table 1.

A statistically significant difference in postexertion CS concentration and its percentage change was shown in the general characteristics of the patients depending on reaching CE during the 12-month follow-up. After sub- and maximum physical exertion, a clear decrease in CST concentration was observed in the group in which CE was noted. The general characteristics of the study group with a division depending on the occurrence of CE for the 24-month period are presented in Table 2.

In the present study, no statistically significant difference in plasma CST concentration was found in patients depending on diabetes, atrial fibrillation, heart failure etiology, or the presence of an implantable cardioverter-defibrillator, as shown in Table 3.

Similarly, the linear regression model did not reveal any significant impact of administered medications on baseline, postexertion, and change (*δ*%) of catestatin plasma concentration.

Plasma CST concentration before exertion was statistically insignificantly lower in the study group compared to the control group. The exertion during the ergospirometry test did not reveal differences in the catestatin concentration between the study group and the control group. A number of significant differences were observed between the study group and the control group, including age, LVEF, TAPSE, RDW, BMI, creatinine, troponin T, NT-proBNP, and ergospirometric parameters.

Patients who reached the composite endpoint during the 24-month follow-up were characterized by statistically significantly lower levels of catestatin, both assessed before exertion and after sub- and maximum physical exertion. After ergospirometry, a clear decrease in the catestatin concentration was observed in the CE group. Moreover, the group of patients in whom CE was reported more often took the drug from the group of vitamin K antagonists and had a higher creatinine concentration.

Spearman's rank correlation showed significant negative correlations between catestatin assessed after sub- and maximal physical exertion in the study group of patients and biomarkers with an established position in modern cardiology (CST post vs. NT-proBNPpre, *r* = −0.18; CST post vs. TnTpre, *r* = −0.33; CST post vs. TnT post, *r* = −0.24) and no significant correlation between traditional diagnostic and prognostic markers used in HF, such as hs-CRP and NT-proBNP, and plasma CST concentration evaluated before physical exertion. A significant negative correlation was also observed between CST *δ*% and V02 peak (*r* = −0.23).

In a one-way analysis carried out using the Cox proportional hazard model over a 24-month period, a statistically significant effect of all assessed CST concentrations (CST pre, CST post, and CST *δ*%) on the occurrence of CE was demonstrated. The effect of other assessed factors turned out to be statistically insignificant. The above results are presented in Table 4.

In a logistic regression analysis carried out over a 12-month follow-up period, no statistically significant effect of CST on CE was shown. In a 24-month follow-up, where the baseline catestatin concentration was compared with other recognized prognostic factors, baseline CST was the only tested parameter to show statistical significance in CE prediction (Table 5).

Table 6 shows the cutoff points determined based on the ROC curves for selected parameters.

**Figure 1:**
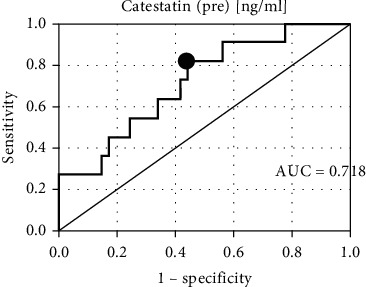
The receiver operating characteristic curve for catestatin (pre) at 24 months of follow-up.

**Figure 2:**
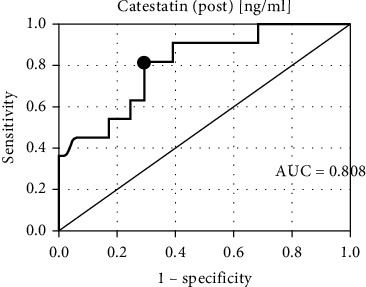
The receiver operating characteristic curve for catestatin (post) at 24 months of follow-up.

Both preexertion catestatin and postexertion catestatin proved to be important CE predictors (Figure 1 and Figure 2).

**Figure 3:**
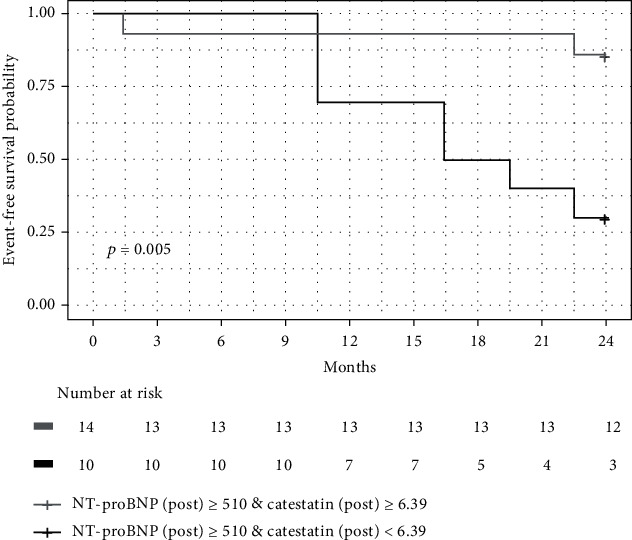
The Kaplan-Meier event-free survival curves in groups with NT-pro BNP (post) ≥cutoff point and CST (post) ≥cutoff point vs. NT-proBNP ≥cutoff point and CST (post) < cutoff point for 24 months of follow-up.

The cutoff values were then applied to the Kaplan-Meier event-free survival curves, where significant differences in event-free survival were observed between groups with NT-proBNP (post) ≥ cutoff point and CST (post) ≥ cutoff point vs. NT-proBNP ≥cutoff point and CST (post) < cutoff point (Figure 3).

## 5. Discussion

A review of the current literature indicates that this is the first paper describing the prognostic significance of CST in the group of patients with HFrEF and the second in the group of patients with CHF; the first to assess the prognostic value of CST in the group of hemodynamically stable patients with HF; and the first to assess the change in catestatin concentration under the influence of short-term physical exertion in humans.

The decision to assess baseline and postexercise CST concentration in the control group was made because of the lack of sufficient information in the literature on normal ranges in the healthy population. Most studies published up to date evaluated CST in asymptomatic patients with risk factors for CHF development and in patients with less severe forms of symptomatic heart failure (NYHA class I and II). The present study would not be complete without the information on CST concentration in healthy individuals. We have also suspected that the difference between healthy people and stable CHF patients on optimal treatment might be minimal or nonexistent which proved to be the case. There is, however, a statistically significant difference in age between the patients and the control group in the present study, which may influence the results. In investigating patients with heart failure, finding the appropriate age-matched control group always poses a significant challenge. It is mostly the result of the high prevalence of both cardiovascular risk factors and asymptomatic hypertension or hyperlipidemia in age-matched individuals. The decision was made to include only completely healthy patients that ultimately resulted in significantly younger control than the study group, which certainly is a limitation of the study. The assessment of CST changes after the cardiopulmonary stress test was included in the protocol, since exertion challenge in the context of the catestatin plasma concentration has never been done neither in patients with heart failure nor in healthy volunteers. The assumption was made that due to compensation mechanisms typically involved in the pathogenesis of heart failure (namely: upregulation of sympathetic nervous system or renin-angiotensin-aldosteron axis activation), CST changes in response to exercise may be completely different in patients than in healthy volunteers. The omission of this part of the study protocol would make the results incomplete and would make a difficult thorough interpretation of the results.

It should be emphasized that the study group of stable patients was carefully selected, which is confirmed by a very low mortality rate -4% and a very low frequency of unplanned hospitalizations, which was recorded only in 17% of the study group in the 24-month follow-up period. The plasma concentrations of traditional diagnostic and prognostic markers used in heart failure assessed before physical exertion are another proof of the homogeneity of the study group. The NT-proBNP concentration was 441.5 pg/ml (181-1080), TnT concentration 0.012 *μ*g/l (0.008-0.016), and hs-CRP concentration 1.10 mg/l (0.75-2.46), while hemoglobin and RDW concentration 14.7 g/dl (14.05-15.4) and 13.55% (13-14.1), respectively, and V02max/VO2peak in the group of patients amounted to 18.01 ± 4.96 l/kg/min.

The only one currently published report assessing the prognostic significance of catestatin is the study by Zhu et al. The authors assessed the CST concentration in various HF phases and the diagnostic utility of CST as a potential biomarker for detecting asymptomatic HF in stage B according to the American Heart Association (AHA). In a group of 300 patients (stage B—*n* = 76, age 68.58 ± 8.63, LVEF 54.95 ± 9.82%), it was shown that the concentration of CST decreased from stage A, through stage B, to stage C. The CST cutoff value for detection of stage B HF was 19.73 ng/ml with 90% sensitivity (higher in this study than for BNP) and a specificity of 50.9%. The CST concentration did not correlate with BNP concentration (*r* = 0.107, *p* = 0.150). According to the authors, asymptomatic patients in HF stage B would benefit most from regular observation and therapeutic intervention; as observed in this group, a decrease in catestatin concentration may precede full-blown HF [17].

Comparing the group from our study vs. Zhu et al.—stage C (symptomatic patients), significant differences should be emphasized as to the optimal pharmacological treatment (beta-blocker 100% vs. 86.2%, ACEi or sartan 100% vs. 72.4, spironolactone or eplerenone 100% vs. no data) and the fact that all patients from our study were hemodynamically stable patients, without signs of exacerbation of HF symptoms, whereas AHA stage C encompasses a wide range of symptomatic patients. These 2 factors seem to have a significant impact on the difference in the obtained results. Similar to the presented study, Zhu et al. also failed to show any correlation between the NT-proBNP concentration and CST. In stable patients included in the present study, the natriuretic peptide concentration was relatively low reflecting the good hemodynamic status of the observed group. The lack of correlation between baseline NT-proBNP and CST may be a result of different mechanisms underlying the production and excretion of both markers. In the present study, statistically significant correlations were observed between postexertion NT-proBNP and CST and between pre- and postexertion troponin and CST. Yet, these correlations were weak; therefore, this phenomenon requires further analysis of large groups of patients to draw any valuable conclusions.

Another study aimed at assessing the CST diagnostic potential in stable and exacerbated HF patients was the analysis by Liu et al. The authors observed no significant differences in the CST levels among NYHA I, NYHA II, and the control group patients; however, the plasma catestatin levels in patients with NYHA III and IV were significantly higher. In the group of patients with medium and severe HF (NYHA III or IV), no significant differences were observed in the CST concentration depending on sex, HFpEF, or HFrEF. Yet, in the group of ICM vs. NICM patients, this difference was significant (*p* = 0.002). The multivariate analysis showed that the NYHA class, ICM, and eGFR independently predicted LogCST in plasma that was independent of the BNP concentration [18].

The result of the above study regarding the lack of a significant relationship between the concentration of natriuretic peptide and catestatin is identical to the presented paper. Liu et al. similarly to the presented analysis showed no significant difference in the plasma catestatin concentration between hemodynamically stable patients (NYHA I and NYHA II) and a group of healthy volunteers. The lack of difference in the plasma CST concentration in the presented analysis depending on the HF etiology may be due to the low ICM percentage in the HF group. Liu et al. showed a significant difference in the CST concentration between the NYHA class II vs. NYHA class III, which is in contrast to the presented analysis. This finding is difficult to interpret as it may be the result of the liberal inclusion criteria to the study group developed by the cited authors. Compared to the presented analysis, the study group of Liu et al. is heterogeneous, since both patients are with HFrEF and HFpEF, hemodynamically stable, and those during the exacerbation of the disease were qualified; moreover, the pharmacological treatment of patients seems to be suboptimal.

In contrast to the presented findings, Peng et al., investigating the heterogenous group of patients with CHF during the 52-month follow-up, observed a significantly higher baseline catestatin concentration in patients who died of all and cardiovascular causes vs. the group with no endpoint recorded. According to Cox multifactorial regression, the plasma catestatin concentration proved to be an independent predictor of death from cardiological reasons, whereas in respect of all-cause deaths, the predictive value of CST was critically insignificant (*p* = 0.051) [19].

Similar to the above-cited Liu et al. paper, the heterogeneity of the group observed in Peng et al. study makes the interpretation of these findings relatively difficult. The authors included in their analysis patients with completely different pathophysiology of heart failure—those with reduced and preserved left ventricular ejection fraction. Although the prognosis for both HF groups may well be equally serious, the reaction for administered treatment is often different reflecting various pathomechanisms involved in the natural history of the disease [20].

The presented study aimed to analyse the prognostic significance of both the CST baseline plasma concentration and concentration after exertion. The ROC analysis indicating the cutoff values of CST-post and constructed accordingly Kaplan-Meier curves of event-free survival revealed that simultaneous assessment of NT-proBNP and CST postexertion allows for the identification of patients with a more severe course of the disease. These individuals may benefit from systematic follow-up and may be candidates for more aggressive treatment. Certainly, evaluating the biochemical markers after sub- or maximal exercise is not always feasible as not every HF patient requires such testing. However, taking into account that the clinical evaluation before a heart transplant usually encompasses a cardiopulmonary exercise stress test, an additional biomarker assessment should not pose any inconvenience. Even in this population of severely ill patients, stable individuals may not constitute a uniform group, and the identification of those with the poorest prognosis could be helpful.

The limitations of the presented study are as follows:

- Relatively small studied groups
- No assessment of possible changes in chromogranin A proteolysis disorders in the studied populations [21]
- A significant difference in age between the study and control group

## 6. Conclusions

1. The plasma concentration of catestatin before and after physical exertion is a valuable prognostic parameter in predicting the all-cause death and unplanned hospitalization in the group of patients with HFrEF in the 2-year follow-up
2. Catestatin has no diagnostic value in the diagnosis of patients with compensated heart failure with a reduced left ventricular ejection fraction
3. The prognostic value of catestatin in the group of patients with HFrEF becomes increasingly important in long-term follow-up
4. Traditional diagnostic and prognostic markers used in heart failure, such as hs-CRP, TnT, and NT-proBNP, are not related to the plasma concentration of catestatin evaluated before the physical exertion

## Figures and Tables

**Table 1:** Study group vs. control group.

	Control group (*n* = 24)	Study group (*n* = 52)	*p*
Age (years)^∗^	35.7 ± 12.3	51.6 ± 9.2	**<0.0001**
Men	**70.8%**	90.4%	0.04
EF (%)^∗^	64.8 ± 7	28.7 ± 7.5	<0.0001
TAPSE (mm)^∗^	24.8 ± 3	20.8 ± 3.9	<0.0001
BMI (kg/m^2^)^∗^	23.5 ± 3	28.7 ± 5.0	<0.0001
GFR >60	100%	84.6%	0.05
Creatinine (mg/dl)^∗∗^	**0.86 (0.73-0.94)**	**0.96 (0.85-1.08)**	**0.003**
Hgb (g/dl)^∗∗^	14.75 (13.3-15.4)	14.7 (14.05-15.4)	0.69
HCT (%)^∗∗^	43.15 (39.87-44.15)	43.03 (41.5-44.72)	0.30
PLT (tys/mm^3^)^∗∗^	**236 (205.75-248.5)**	**205 (165-237.25)**	**0.02**
RDW (%)^∗∗^	**12.95 (12.5-13.4)**	**13.55 (13-14.1)**	**0.001**
WBC (tys./mm^3^)^∗∗^	**5.42 (4.84-6.10)**	**7.46 (5.95-8.65)**	**<0.0001**
Neutrocytes (%)^∗∗^	**51.8 (49.95-54.37)**	**56.15 (51.4-64.15)**	**0.0032**
Hs-CRP (mg/l)^∗∗^	0.92 (0.545-1.435)	1.105 (0.755-2.457)	0.86
TnTpre (*μ*g/l)^∗∗^	**0.005 (0.004-0.007)**	**0.012 (0.008-0.016)**	**<0.0001**
TnT post (*μ*g/l)^∗∗^	**0.005 (0.004-0.006)**	**0.012 (0.009-0.018)**	**<0.0001**
TnT *δ*% ^∗∗^	**23.8 (14.42-31.87)**	**12.3 (4.57-18.35)**	**0.0003**
NT-proBNPpre (pg/ml)^∗∗^	**33 (23.5-51)**	**441 (181-1080)**	**<0.0001**
NT-proBNP post (pg/ml)^∗∗^	**44.5 (26.75-60.75)**	**1153 (208-1280)**	**<0.0001**
NT-proBNP *δ*%^∗∗^	0 (0-0)	0 (0-8.3)	0.35
V02max/VO2peak (l/kg/min)^∗^	37.17 ± 7.52	18.01 ± 4.96	**<0.0001**
VE/VC02 (%)^∗^	30.13 ± 3.84	35.40 ± 7.27	**0.0001**
OUES^∗^	2.67 ± 0.86	1.90 ± 0.75	**0.0002**
RER^∗^	1.21 ± 0.10	1.03 ± 0.18	**<0.0001**
CST pre (ng/ml)^∗∗^	16.6 (14.75-22.20)	15.95 (13.89-18.81)	0.12
CST post (ng/ml)^∗∗^	9.26 (6.11-140.23)	7.04 (4.97-11.08)	0.13
CST *δ*%^∗∗^	-86,56 (-85,8-126,5)	-148 (71-181)	0,08

EF: ejection fraction; TAPSE: tricuspid annular plane systolic excursion; BMI: body mass index; GFR: glomerular filtration rate; Hgb: hemoglobin; HCT: hematocrit; PLT: platelets; RDW: red cell distribution width; WBC: white blood cells; hs-CRP: high-sensitivity C-reactive protein; TnTpre: cardiac troponin T (concentration assessed before physical exertion); TnTpost: concentration assessed immediately after physical exertion; TnT *δ*%: variability of TnT concentration under the influence of physical effort; NT-proBNP: N-terminal proBNP; VO2max: maximal oxygen consumption; V02peak: peak oxygen uptake; OUES: oxygen uptake efficiency slope; RER: respiratory exchange ratio; CST: catestatin. Statistically significant results are marked in bold. The results in the tables are presented as follows: ^∗^means ± standard deviation. ^∗∗^medians (lower quartile–upper quartile).

**Table 2:** Basic characteristics of the study group depending on end-point occurrence in the 24-month follow-up.

	All (*n* = 52)	End point (*n* = 11; 21.15%)	No end point (*n* = 41; 78.85%)	*p*
Age (years)^∗^	51.6 ± 9.1	52 ± 9.5	51.5 ± 9.2	0.87
Men	90.4%	100%	87.8%	0.57
EF (%)^∗^	28.7 ± 7.5	27.9 ± 7.6	28.9 ± 7.6	0.72
TAPSE (mm)^∗^	20.8 ± 3.9	19 ± 3.9	21.32 ± 3.8	0.08
Ischaemic aetiology	30.8%	18.18%	34.2%	0.47
BMI (kg/m^2^)^∗^	28.7 ± 5.0	27.4 ± 6.2	29.1 ± 4.7	0.31
NYHA class III	25%	18.18%	26.8%	0.71
DMT2	28.9%	36.36%	26.8%	0.71
Insulin	7.7%	9.1%	7.3%	1.00
AHT	34.6%	9.1%	41.5%	0.07
AF	38.5%	36.4%	68.3%	0.08
ICD	51.9%	63.6%	48.8%	0.50
GFR>60	84.6%	81.8%	85.4%	1.00
Creatinine (mg/dl)^∗∗^	*0.96 (0.85-1.08)*	*1.03 (0.96-1.22)*	*0.99 (0.81-1.05)*	*0.04*
Hgb (g/dl)^∗∗^	14.7 (14.05-15.4)	14.8 (14.1-14.95)	14.7 (14.1-15.4)	0.85
HCT (%)^∗∗^	43.55 (41.5-44.7)	43.5 (42.65-45)	43.6 (40.3-44.7)	0.37
PLT (tys./mm^3^)^∗∗^	205 (165-237.2)	221 (149.5-268.5)	201 (172-236)	0.99
RDW (%)^∗∗^	13.55 (13-14.1)	13.7 (13.2-14.6)	13.5 (13-14.1)	0.50
WBC (tys./mm^3^)^∗∗^	7.46 (5.95-8.65)	7.7 (6.11-8.76)	7.4 (5.68-8.41)	0.52
Neutrocytes (%)^∗∗^	56.15 (51.4-64.1)	56.3 (51.9-65.85)	56 (51.1-62)	0.82
Hs-CRP (mg/l)^∗∗^	1.10 (0.75-2.46)	2.16 (0.81-2.27)	0.96 (0.71-2.72)	0.90
TnTpre (*μ*g/l)^∗∗^	0.012 (0.008-0.016)	0.014 (0.01-0.02)	0.011 (0.008-0.016)	0.12
TnT post (*μ*g/l)^∗∗^	0.012 (0.009-0.018)	0.013 (0.01-0.019)	0.012 (0.008-0.016)	0.30
TnT *δ*%^∗∗^	12.3 (4.57-18.35)	13.5 (7.5-17.65)	11.1 (3.9-18.2)	0.51
NT-proBNPpre(pg/ml)^∗∗^	441.5 (181-108)	758 (472.5-112)	373 (175-705)	0.11
NT-proBNPpost (pg/ml)^∗∗^	442 (208-1280)	955 (512-1380)	403 (189-876)	0.10
NT-proBNP *δ*%^∗∗^	0 (0-8,3)	0 (-6.25-1.9)	0 (0-8.3)	0.28
V02max/VO2peak (l/kg/min)^∗^	18.01 ± 4.96	18.22 ± 3.73	17.95 ± 5.28	0.88
VE/VC02 (%)^∗^	35.40 ± 7.27	37.02 ± 6.82	34.97 ± 7.40	0.41
OUES^∗^	1.90 ± 0.75	1.51 ± 0.51	2.00 ± 0.78	0.05
RER^∗^	1.03 ± 0.18	1.05 ± 0.13	1.02 ± 0.19	0.67
CST pre (ng/ml)^∗∗^	*15.95 (13.89-18.8)*	*14.23 (11.05-15.82)*	*16.86 (14.25-19.46)*	*0.03*
CST post (ng/ml)^∗∗^	*7.04 (4.97-11.08)*	*4.81 (2.20-6.25)*	*7.82 (5.81-63.48)*	*0.002*
CST *δ*%^∗∗^	*-148 (71-181)*	*-254 (161-314)*	*-124 (-71-164)*	*0.002*
ACEi	78.9%	72.7%	80.5%	0.68
ARB	23.1%	27.3%	22%	0.70
Statin	82.7%	90.9%	80.5%	0.66
Beta-bloker	100%	100%	100%	-
ASA	40.4%	18.2%	46.3%	0.17
Digoxin	7.7%	9.1%	7.3%	1.00
Spironolakton	44.2%	54.6%	41.5%	0.51
Eplerenon	59.6%	54.6%	61%	0.74
Iwabradyna	11.5%	27.3%	7.3%	0.10
VKA	*34.6%*	*72.7%*	*24.4%*	*0.0048*
NonVKA	5.8%	0%	7.3%	1.00
Amiodaron	7.7%	18.2%	4.9%	0.19
Furosemid	25%	27.3%	24.4%	1.00
Torasemid	36.5%	54.6%	31.7%	0.18
Hydrochlorotiazyd	15.4%	0 (0%)	19.5%	0.18

EF: ejection fraction; TAPSE: tricuspid annular plane systolic excursion; BMI: body mass index; DMT2: diabetes mellitus type 2; AHT: atrial hypertension; AF: atrial fibrillation; ICD: implantable cardioverter-defibrillator; GFR: glomerular filtration rate; Hgb: hemoglobin; HCT: hematocrit; PLT: platelets; RDW: red cell distribution width; WBC: white blood cells; hs-CRP: high-sensitivity C-reactive protein; TnTpre: cardiac troponin T (concentration assessed before physical exertion); TnTpost: concentration assessed immediately after physical exertion; TnT *δ*%: variability of TnT concentration under the influence of physical effort; NT-proBNP: N-terminal proBNP; VO2max: maximal oxygen consumption; V02peak: peak oxygen uptake; OUES: oxygen uptake efficiency slope; RER: respiratory exchange ratio; CST: catestatin; ACEi: angiotensin-converting-enzyme inhibitors; ARB: angiotensin II receptor blockers; ASA: acetylsalicylic acid; VKA: vitamin K antagonists; nonVKA: nonvitamin K antagonist. Statistically significant results are marked in italics. The results in the tables are presented as follows: ^∗^means ± standard deviation. ^∗∗^medians (lower quartile–upper quartile).

**Table 3:** Differences in plasma CST concentration (ng/ml) depending on the clinical conditions of the patients.

	DMT2 (*N* = 15)			No DMT2 (*N* = 37)			
	Mean ± SD	Median	Quartiles	Mean ± SD	Median	Quartiles	*p*
CST pre	16.88 ± 4.17	16.6	14.59-18.29	16.43 ± 4.88	15.4	13.66-19.46	0.64
CSTpost	49.41 ± 90.15	6.62	5.19-10.32	47.51 ± 84.88	7.49	5.03-11.98	0.70
CST *δ*%	135.13 ± 411.93	-61.51	-64.53-31.79	176.63 ± 510.16	-56.6	-64.26-43.47	0.49
	AF (*N* = 20)			SR (*N* = 32)			
CST pre	15.72 ± 5.58	15.21	13.44-17.46	17.09 ± 3.97	16.73	14.18-19.28	0.29
CSTpost	55.73 ± 91.7	6.30	4.19-45.73	43.26 ± 82.6	7.64	5.76-10.51	0.33
CST *δ*%	236.3 ± 573.28	-62.10	-72.09-100.02	119.88 ± 415.52	-56.03	-61.68-41.35	0.21
	DCM (*N* = 36)			ICM (*N* = 16)			
CST pre	16.7 ± 5.09	15.95	14.01-18.59	16.24 ± 3.6	16.16	13.14-19.28	0.80
CSTpost	48.9 ± 89.39	6.89	4.93-10.2	46.17 ± 78.93	7.66	5.34-25.38	0.71
CST *δ*%	159.99 ± 496.19	-59.98	-65.31-46.1	13.14-19.28	-58.41	-63.09-48.73	0.50
	ICD (*N* = 27)			NO ICD (*N* = 25)			
CST pre	16.1 ± 5.1	16.52	13.07-18.2	17.06 ± 4.16	15.64	14.25-19.22	0.50
CSTpost	63.37 ± 96.47	7.8	5.21-105.22	31.51 ± 70.13	6.76	5.03-9.45	0.33
CST *δ*%	261.72 ± 558.17	-55.37	-64.54-397.91	59.83 ± 361.36	-60.25	-63.75-51.6	0.20

DMT2: type 2 diabetes; AF: atrial fibrillation; SR: sinus rhythm; DCM: dilated cardiomyopathy; ICM: ischemic cardiomyopathy; ICD: implantable cardioverter-defibrillator.

**Table 4:** One-factor analysis carried out using the Cox proportional hazard model over a 24-month follow-up period.

—	*p*	HR	(-95%; 95% confidence interval)
CST pre	0.01	0.84	(0.73; 0.96)
CST post	0.026	0.76	(0.59; 0,97)
CST *δ*%	<0.001	1.0041	(1.0017; 1.0065)
Age (years)	0.94	1	(0.94; 1.07)
Men	0.98	1	(1; 1)
EF	0.71	0.99	(0.91; 1.07)
TAPSE	0.12	0.89	(0.76;1.03)
Ischaemic aetiology	0.38	0.50	(0.11; 2.33)
BMI	0.29	0.93	(0.82; 1.06)
NYHA III	0.58	0.65	(0.14; 3.01)
Insulin	0.91	1.13	(0.14; 8.80)
AF	0.09	2.88	(0.84; 9.85)
ICD	0.45	1.61	(0.47; 5.51)
Hgb	0.86	0.96	(0.58; 1.57)
RDW	0.13	1.27	(0.93; 1.93)
WBC	0.61	1.08	(0.80; 1.47)
Neutrocytes	0.79	1.01	(0.94; 1.98)
TnTpre	0.31	1.28	(0.23; 0.72)
NT-proBNPpre	0.85	1.00	1
NT-proBNP post	0.83	1.00	1
NT-proBNP *δ*%	0.18	0.96	(0.90; 1.02)
V02max/V02peak	0.85	1.01	(0.90; 1.14)
OUES	0.06	0.41	(0.16; 1.02)
Ve/VCo2	0.44	1.03	(0.96; 1.11)
Hs-CRP	0.61	0.92	(0.65; 1.28)

**Table 5:** Multivariate analysis carried out using the logistic regression model over a 24-month observation period.

	*p*	HR	(-95%; 95% confidence interval)
CST pre	0.04	0.74	(0.56; 1.12)
RDW %	0.43	1.24	(0.73; 2.11)
NT-proBNP(pre) pg/ml	0.34	1	(1; 1)
Hs-CRP mg/l	0.79	0.93	(0.53; 1.63)
TnT(pre) × 100	0.44	1.28	(0.68; 2.38)
OUES	0.11	0.25	(0.05; 1.38)

**Table 6:** Determined cutoff points based on the ROC curves for NT-proBNPpre, NT-proBNP post, CST pre, CST post.

Parameter	Cut point	Sensitivity at cut point	Specificity at cut point	Direction	AUC	*p* ^∗^
NT-proBNP (pre) (pg/ml)	455	81.82%	60.98%	Positive	0.659	0.08
NT-proBNP (post) (pg/ml)	510	81.82%	63.41%	Positive	0.662	0.07
Catestatin (pre) (ng/ml)	16.52	81.82%	56.10%	Negative	0.718	*0.012*
Catestatin (post) (ng/ml)	6.39	81.82%	70.73%	Negative	0.808	*<0.001*

^∗^DeLong's method (null hypothesis: AUC =0.5).

## Data Availability

All data used to support the finding of this study are available from the corresponding author upon request.
